# Ionic Liquid-Supported Photocatalysts: A Reusable Environmentally Friendly Oxidation Reaction System That Uses Air and Light

**DOI:** 10.3390/ijms24087141

**Published:** 2023-04-12

**Authors:** Shinichi Koguchi, Haruto Fujita, Yuga Shibuya

**Affiliations:** 1 Department of Chemistry, Tokai University, 4-1-1 Kitakaname, Hiratsuka 259-1292, Kanagawa, Japan; 2 Graduate School of Science and Technology, Tokai University, 4-1-1 Kitakaname, Hiratsuka-shi 259-1292, Kanagawa, Japan

**Keywords:** ionic liquids, ionic liquid-supported catalysts, photocatalysts, cleavage of vicinal diol

## Abstract

Ionic liquids are used in various fields due to their unique physical properties and are widely utilized as reaction solvents in the field of synthetic organic chemistry. We have previously proposed a new organic synthetic method in which the catalyst and reaction reagents are supported on ionic liquids. This method has various advantages, such as the ability to reuse the reaction solvent and catalyst and its facile post-reaction treatment. In this paper, we describe the synthesis of an ionic liquid-supported anthraquinone photocatalyst and the synthesis of benzoic acid derivatives using this system. This synthesis of benzoic acid derivatives via the cleavage of vicinal diols by an ionic liquid-supported anthraquinone photocatalyst is an environmentally friendly process, and furthermore, it has a simple post-reaction process, and the catalyst and solvent can both be reused. To the best of our knowledge, this is the first report on the synthesis of benzoic-acid derivatives via the cleavage of vicinal diols using light and an ionic-liquid-supported catalyst.

## 1. Introduction

Ionic liquids have many unique properties, including non-volatility, good thermal and electrical conductivities, large polarities and good thermal stabilities. Therefore, ionic liquids have attracted considerable attention from the scientific community. To date, our research has focused on the use of ionic liquids as organic reaction solvents. The advantages of using an ionic liquid as a reaction solvent are that they are non-volatile and, thus, are environmentally friendly, they can be reused, and they have high thermal stability, so they are suitable for high-temperature reactions [1,2,3,4]. We have developed ionic liquid-supported catalysts and reagents to construct more environmentally friendly organic reaction systems. We have previously reported the synthesis of ionic liquid-supported 18-crown-6 ether [5], an ascorbate-based ionic liquid [6], ionic liquid-supported benzyl chloride [7] for use in Huisgen click chemistry, an ionic liquid-supported hypervalent iodine reagent [8] for alcohol oxidation, ionic liquid-supported 1,3-dimethylimidazolidin-2-one [9] for halogenation reactions, and ionic liquid-supported organotelluride [10,11] for the oxidation of thiol and phosphite esters. The advantages of ionic-liquid supported systems are that they allow easy isolation and purification of the resulting products, and they facilitate the reuse of the catalyst and solvent.

In this study, we attempted to construct a new reaction system in which an anthraquinone photocatalyst is supported on an ionic liquid. There have not been any previous reports on photoreactions that use ionic liquid-supported anthraquinone. This reaction utilizes oxygen from the air and ultraviolet light, whilst the reaction product can be easily extracted and the reaction system can be reused, making this an excellent organic reaction system. The details of the reaction are described below.

Previously, Ito et al. reported a method to prepare benzoic acid via aerobic oxidative cleavage of vicinal diols in the presence of 2-chloroanthraquinone using molecular oxygen as the oxidant [12]. This reaction is an excellent method for converting various diols into benzoic acids utilizing molecular oxygen and light. However, after the reaction is complete, it is necessary to separate the product from the anthraquinone photocatalyst to purify the product, and furthermore, it is difficult to reuse the photocatalyst. To overcome these problems, we investigated the preparation of ionic liquid-supported anthraquinone and its application in the oxidative cleavage of vicinal diols to form benzoic acids.

## 2. Results and Discussion

**Scheme 1:**
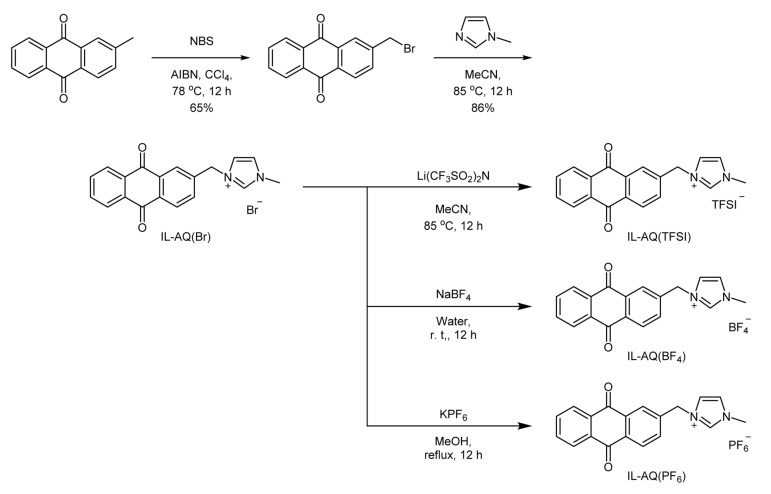
Synthesis of ionic liquid-supported anthraquinone.

As shown in Scheme 1, we initially focused on the synthesis of the ionic liquid-supported anthraquinone. 2−Methylanthraquinone was converted to 2−(bromomethyl) anthracene using *N*−bromosuccinimide (NBS) and azobisisobutyronitrile (AIBN) as a radical initiator under thermal conditions. 2−(Bromomethyl) anthracene was converted to an ionic liquid-supported anthraquinone (IL-AQ(Br)), with bromine as the counter anion, via reaction with methylimidazole. IL-AQ(Br) was converted into multiple different ionic liquid-supported anthraquinones via ion exchange with various inorganic salts. These resulting ionic liquid-supported anthraquinones, IL-AQ(Br), IL-AQ(TFSI) and IL-AQ(BF_4_), with Br, TFSI and BF_4_ counter anions were water soluble. IL-AQ(Br) was also subsequently converted into a hydrophobic ionic liquid-supported anthraquinone, IL-AQ(PF_6_), which was insoluble in low-polarity organic solvents and water. The structures of the ionic liquid catalysts were confirmed using ^1^H and ^13^C NMR spectroscopy.

We investigated the catalytic aerobic photooxidative cleavage reaction of 1−-phenylethane-1,2−diol (1a) using anthraquinone supported on various ionic liquids. An ionic liquid solution of 1-phenylethane-1,2-diol (1a) and the ionic liquid-supported anthraquinone photocatalyst was stirred in an open flask and irradiated with a UV (395 nm) LED lamp at 80 °C. After 15 h, the benzoic acid product was isolated via extraction with diethyl ether. The yields are compiled in Table 1.

According to previous studies, a reaction system in which the counter anion of the ionic liquid-supported catalyst and the counter anion of the ionic liquid solvent are the same yields good results [3,5,6,7,8].

Therefore, in this investigation, we examined a reaction system in which the counter anion of the ionic liquid-supported catalyst and the counter anion of the ionic liquid were the same (Table 1, entries 1–4). These results generally produced good yields of the benzoic acid product. However, the reaction systems with the counter anions PF_6_, TFSI and Br (Table 1, entries 1, 2 and 4) gave slightly decreased yields with respect to the BF_4_ system (Table 1, entry 3). After completion of the reaction, the PF_6_, TFSI and Br ionic liquids were observed to be slightly discolored and black. It is thought that these ionic liquids or ionic liquid-supported catalysts are probably decomposed by the UV (395 nm) light and temperature. On the other hand, in the reaction system that utilizes the IL-AQ(BF_4_) ionic liquid-supported catalyst and the (bmim)BF_4_ ionic liquid solvent (Table 1, entry 3), decomposition of the ionic liquid was not observed and the product was obtained in a good yield. Next, we investigated reactions using different ionic liquid-supported catalysts in the (bmim)BF_4_ solvent and found that the yields decreased (Table 1, entries 5–7). These reactions were all examples in which the counter anion of the ionic liquid-supported catalyst and the counter anion of the ionic liquid used as the solvent were different. It is likely that the decreased yields arose because the ionic liquid-supported catalyst did not dissolve well in the ionic liquid. Furthermore, in the reaction using free anthraquinone as a photocatalyst, a significant decrease in yield was not observed, albeit that anthraquinone was extracted from the reaction mixture together with the product (Table 1, entry 8). It was, therefore, necessary to separate the product and anthraquinone from the extraction solvent, and the anthraquinone could not be easily reused. The difficulties in reusing anthraquinone in this reaction system are predominantly associated with the very low polarity of free anthraquinone, which diminishes its persistency in the ionic-liquid phase. Barely any reaction was observed when the photocatalyst and the UV light source (395 nm) were omitted from the system (Table 1, entries 9 and 10), thus confirming that this reaction requires a photocatalyst and UV light irradiation at 395 nm. A decrease in yield was observed when the amount of photocatalyst was further decreased (Table 1, entry 11). Overall, it was found that the most suitable conditions for this reaction are the use of the IL-AQ(BF_4_) photocatalyst (0.1 eq.) and (bmim)BF_4_ ionic liquid solvent in the presence of UV light (395 nm) irradiation (Table 1, entry 3).

Next, we investigated the scope of the vicinal diols tolerated by our aerobic oxidative cleavage reaction under the established optimal reaction conditions (Table 2). In all cases, the reaction proceeded to give the expected benzoic acid derivatives in good to excellent yields. An exception to this was the diol with a para phenyl acetate group, which gave the corresponding benzoic acid derivative in only a 95% yield. (When 4-(1,2-dihydroxyethyl) phenyl acetate was reacted, the acetoxy benzoic acid and a small amount of hydroxyacetophenone derivative were obtained. Hydroxyacetophenone is an intermediate of benzoic acid. Therefore, only this reaction was purified using column chromatography.

Finally, we examined the reusability of the catalyst for the aerobic oxidative cleavage of vicinal diols. The oxidative cleavage of 1-phenylethane-1,2-diol (1a) was conducted, and once the reaction was complete, the benzoic acid product was extracted with diethyl ether (Table 3). Subsequently, more 1-phenylethane-1,2-diol (1a) substrate was added, and the aerobic oxidative cleavage reaction was conducted once more. It was found that the remaining (bmim)BF_4_ solution containing the IL-AQ(BF_4_) photocatalyst could be reused at least five times in subsequent reactions without a detectable deterioration in performance (Table 3, entry 1). However, it was harder to efficiently obtain the product over multiple cycles when using (bmim)PF_6_ as the solvent and IL-AQ(PF_6_) as the ionic liquid-supported catalyst (Table 3, entry 2). This reaction system turned black as it was repeatedly recycled. Furthermore, the reaction using (bmim) TFSI as the solvent and IL-AQ(TFSI) as the ionic liquid-supported catalyst could only be recycled three times (Table 3, entry 3). Following the fourth cycle, when we added diethyl ether to the ionic liquid and attempted to extract the desired product, the ionic liquid and diethyl ether did not separate (We also unsuccessfully tried to extract using *n*-hexane). It was clear that the ionic liquid and the ionic liquid-supported catalyst had been decomposed by heat and ultraviolet light. The most suitable system for recycling the reaction was the combination of the (bmim)BF_4_ ionic liquid and the IL-AQ(BF_4_) ionic liquid-supported catalyst.

**Scheme 2:**
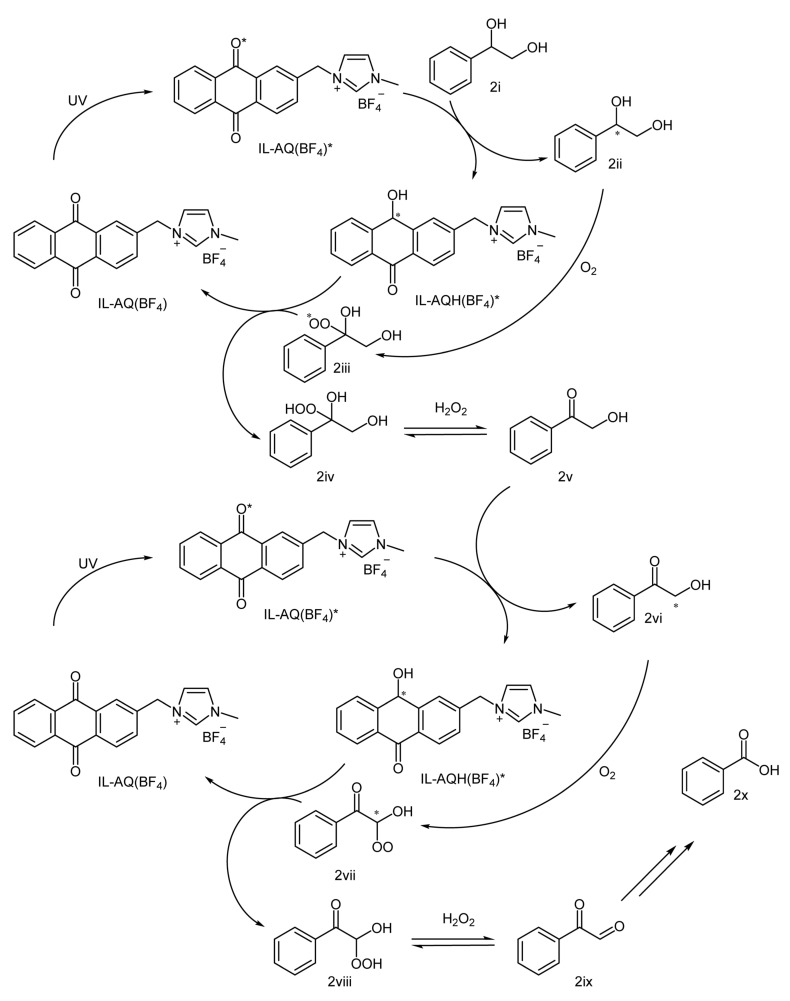
Proposed reaction mechanism.

A possible catalytic cycle is proposed in accordance with previous reports (Scheme 2) [12]. The proposed reaction mechanism begins with an excited molecule of the ionic liquid-supported anthraquinone (IL-AQ(BF_4_)*, abstracting a benzylic hydrogen atom from the diol (2i), generating a peroxyl radical, (2iii), following a reaction with O_2_. This species then reacts with an IL-AQH(BF_4_) radical to produce hydroperoxide (2iv). Next, hydroperoxide (2iv) is converted to hydroxy acetophenone derivative (2v), and another hydrogen atom is abstracted by the re-excited ionic liquid-supported anthraquinone species (IL-AQ(BF_4_)*). The generated peroxyl radical (2vii) receives a hydrogen atom from the IL-AQH(BF_4_) radical and is converted to a neutral hydroperoxyl molecule (2viii). Finally, hydrogen peroxide is eliminated and converted to the target benzoic acid (2x).

## 3. Materials and Methods

### 3.1. General Information Including Important Notices

All reagents and solvents were commercially sourced and of reagent grade and were used without purification. The reactions were monitored using aluminum thin layer chromatography plates with silica gel 60 F254 (Merck, (Darmstadt, Germany)). Column chromatography was performed using silica gel 60 (Kanto Chemical, Japan, Tokyo). ^1^H, ^13^C, and ^125^Te NMR spectra were measured on a Bruker Advance DRX 500 (^1^H: 500 MHz, ^13^C: 125 MHz, ^125^Te: 159 MHz spectrometer. All chemical shifts are reported in parts per million (ppm) relative to TMS (0 ppm for ^1^H), CHCl_3_ (77 ppm, for ^13^C), DMSO (39 ppm for ^13^C), and PhTeTePh (419 ppm in CDCl_3_, 422ppm in DMSO for ^125^Te). Mass analyses were performed using a JEOL AccuTOF LC-plus JMS-T100LP spectrometer (Japan, Tokyo).

### 3.2. Synthesis of the Substrates

#### 3.2.1. General Procedure

Typically, [bmim]BF_4_ solution (3 mL) of vicinal diol (0.30 mmol) and IL-supported anthraquinon IL-AQ(BF_4_) (0.03 mmol) were stirred in an open flask and irradiated using a UV light (395 nm) at 80 °C for 15 h. The resulting mixture was extracted with diethyl ether and evaporated to yield a benzoic acid derivative. The products were identified by comparison of physical and spectral data with published values.

#### 3.2.2. 2-Bromomethyl-9,10-anthraquinone

2-Methyl-9,10-anthoraquinone (0.4450 g, 2.0 mmol) and *N*-bromosuccinimide (0.5321 g, 3.0 mmol) were dissolved in CCl_4_ (60 mL), and the mixture was stirred under reflux for 10 min, then the 2,2′-azobis (isobutyronitrile) (0.0331 g, 0.20 mmol) initiator was added. The mixture was stirred under reflux for 24 h. After the removal of the solvent under reduced pressure, the product was isolated using silica gel column chromatography (hexane-CHCl_3_). Yield: 0.3928 g, 1.3 mmol, 65%; white solid. m. p. = 184–194 °C. ^1^H NMR (500 MHz, CDCl_3_): δ = 4.60 (s, 2H), 7.80–7.83 (m, 3H), 8.29–8.34 (m, 4H). ^13^C NMR (125 MHz, CDCl_3_): δ = 31.6, 127.4, 127.5, 127.7, 128.2, 133.3, 133.6 (2 c), 134.0, 134.4 (2 c), 134.7, 144.3, 182.7, 182.8. HRMS (APCI): *m*/*z* [M − Br + MeCN]^+^ calcd for C_17_H_12_NO_2_: 262.0863; found: 262.0870.

#### 3.2.3. 1-((9,10-Anthraquinon-2-yl)methyl)-3-methyl-1H-imidazol-3-ium Bromide IL-AQ(Br)

2-Bromomethyl-9,10-anthraquinone (1) (0.2008 g, 0.67 mmol) and 1-methylimidazol (0.063 mL, 0.80 mmol) were dissolved in MeCN (5 mL) and the mixture was stirred under reflux for 22 h. After the removal of the solvent under reduced pressure, the product was isolated using silica gel column chromatography (CHCl_3_-MeOH). Yield: 0.2186 g, 0.57 mmol, 86%; yellow solid. m. p. = 208–211 °C ^1^H NMR (500 MHz, DMSO-d_6_): δ = 3.91 (s, 3 H), 5.74 (s, 2 H), 7.82–8.27 (m, 9 H), 9.42 (s, 1 H). ^13^C NMR (125 MHz, DMSO-d_6_): δ = 36.0, 51.1, 122.5, 124.2, 126.6, 126.8 (2 c), 127.6, 132.9, 133.0 (2 c), 133.4, 134.1, 134.7, 134.8, 137.1, 141.4, 182.1, 182.2. HRMS (APCI): *m*/*z* [M − Br]^+^ calcd for C_19_H_15_N_2_O_2_: 303.1128; found: 303.1107.

#### 3.2.4. 1-((9,10-Anthraquinon-2-yl)methyl)-3-methyl-1H-imidazol-3-ium Bis(trifluoromethanesulfonyl) imide IL-AQ (TFSI)

The solution of 1-((9,10-anthraquinon-2-yl)methyl)-3-methyl-1H-imidazol-3-ium Bromide IL-AQ(Br) (0.3262 g, 0.85 mmol) in MeOH (50 mL) was added to lithium bis(trifluoromethansulfonyl) imide (0.2475 g, 0.86 mmol). The resulting mixture was stirred under reflux for 24 h, and then the solvent was removed under vacuum. The product was isolated by silica gel column chromatography (CHCl_3_-MeOH). Yield: 0.2606 g (0.46 mmol 54%); white solid. m. p. = 155–160 °C. ^1^H NMR (500 MHz, DMSO-d_6_): δ = 3.88 (s, 3 H), 5.68 (s, 2 H), 7.77–8.28 (m, 9 H), 9.30 (s, 1 H). ^13^C NMR (125 MHz, DMSO-d_6_): δ = 36.0, 51.2, 118.2 (q, JCF = 320 Hz, 2 c), 122.5, 124.3, 126.6, 126.8 (2 c), 127.6, 133.0 (3 c), 133.4, 134.0, 134.7 (2 c), 137.2, 141.3, 182.1, 182.2. HRMS (APCI): *m*/*z* [M − C_2_F_6_NO_4_S_2_]^+^ calcd for C_19_H_15_N_2_O_2_: 303.1128; found: 303.1115.

#### 3.2.5. 1-((9,10-Anthraquinon-2-yl)methyl)-3-methyl-1H-imidazol-3-ium Tetrafluoroborate IL-AQ (BF_4_)

The solution of 1-((9,10-anthraquinon-2-yl)methyl)-3-methyl-1H-imidazol-3-ium Bromide (2) (0.3257 g, 0.85 mmol) in MeOH (50 mL) was added to sodium tetrafluoroborate (0.0940 g, 0.85 mmol). The resulting mixture was stirred under reflux for 24 h, and then solvent was removed under vacuum. The product was isolated by silica gel column chromatography (CHCl_3_-MeOH). Yield: 0.2788 g, (0.71 mmol, 84%); pale yellow solid. m. p. = 224–229 °C. ^1^H NMR (500 MHz, DMSO-d6): δ = 3.88 (s, 3 H), 5.67 (s, 2 H), 7.76–8.27 (m, 9 H), 9.28 (s, 1 H). ^13^C NMR (125 MHz, DMSO-d6): δ = 36.5, 51.7, 123.0, 124.7, 127.0, 127.3 (2 c), 128.1, 133.5(3 c), 133.9, 134.5, 135.2, 135.3, 137.6, 141.8, 182.6, 182.7. HRMS (APCI): *m*/*z* [M − BF_4_]^+^ calcd for C_19_H_15_N_2_O_2_: 303.1128; found: 303.1126.

#### 3.2.6. 1-((9,10-Anthraquinon-2-yl)methyl)-3-methyl-1H-imidazol-3-ium Hexafluorophosphate IL-AQ (PF_6_)

The solution of 1-((9,10-anthraquinon-2-yl)methyl)-3-methyl-1H-imidazol-3-ium Bromide (2) (0.3815 g, 1.00 mmol) in MeOH (200 mL) was added to potassium hexafluorophosphate (0.1835 g, 1.00 mmol). The resulting mixture was stirred under reflux for 23 h, and then the solvent was removed under vacuum. The product was isolated by silica gel column chromatography (CHCl_3_-MeOH). Yield: 0.3492 g (0.78 mmol, 78%); pale yellow solid. m. p. = 235–239 °C. ^1^H NMR (500 MHz, DMSO-d6): δ = 3.89 (s, 3 H), 5.68 (s, 2 H), 7.76–8.27 (m, 9 H), 9.30 (s, 1 H). ^13^C NMR (125 MHz, DMSO-d6): δ = 36.0, 51.2, 122.5, 124.3, 126.6, 126.9 (2 c), 127.6, 133.0(3 c), 133.5, 134.1, 134.7, 134.8, 137.1, 141.3, 182.1, 182.3. HRMS (APCI): *m*/*z* [M − PF_6_]^+^ calcd for C_19_H_15_N_2_O_2_: 303.1128; found: 303.1117.

#### 3.2.7. Benzoic Acid

Yield: 0.0364 g, (0.30 mmol quant.); white solid. m. p. = 110–112 °C.^1^H NMR (500 MHz, CDCl_3_): δ = 8.13 (d, *J* = 7.2 Hz, 2 H), 7.62 (t, *J* = 7.4 Hz, 1 H), 7.48 (t, *J* = 7.8 Hz, 2 H). ^13^C NMR (125 MHz, CDCl3): δ = 172.3, 134.0, 130.4, 129.4, 128.6. HRMS (DART): *m*/*z* [M + H]^+^ calcd for C_7_H_6_O_2_: 123.0441; found: 123.0452.

#### 3.2.8. 4-Chlorobenzoic Acid

Yield: 0.0463 g, (0.30 mmol 98%); white solid. m. p. = 225–227 °C. ^1^H NMR (500 MHz, DMSO-d_6_): δ = 7.94 (d, *J* = 8.7 Hz, 2 H), 7.57 (d, *J* = 8.6 Hz, 2 H). ^13^C NMR (125 MHz, DMSO-d6): δ = 166.5, 137.8, 131.1, 129.6, 128.8. HRMS (DART): *m*/*z* [M + H]^+^ calcd for C_7_H_5_O_2_Cl: 157.0051; found: 157.0045.

#### 3.2.9. 4-Methylbenzoic Acid

Yield: 0.0488 g, (0.36 mmol quant.); white solid. m. p. = 160–165 °C. ^1^H NMR (500 MHz, CDCl_3_): δ = 8.01 (d, *J* = 8.2 Hz, 2 H), 7.28 (d, *J* = 8.0 Hz, 2 H), 2.44 (s, 3 H). ^13^C NMR (125 MHz, CDCl_3_): δ = 172.0, 144.8, 130.4, 129.4, 126.7, 21.9. HRMS (DART): *m*/*z* [M + H]^+^ calcd for C_8_H_8_O_2_: 137.0598; found: 137.0598.

#### 3.2.10. 4-Acetoxybenzoic Acid

Yield: 0.0517 g, (0.29 mmol 95%); white solid. m. p. = 178–180 °C. ^1^H NMR (500 MHz, CDCl_3_): δ = 8.15 (d, *J* = 8.7 Hz, 2 H), 7.22 (d, *J* = 8.7 Hz, 2 H), 2.34 (s, 3 H). ^13^C NMR (125 MHz, CDCl_3_): δ = 171.1, 169.0, 155.1, 132.0, 126.9, 121.9, 21.3. HRMS (DART): *m*/*z* [M + H]^+^ calcd for C_9_H_8_O_4_: 181.0496; found: 181.0486.

#### 3.2.11. 4-(Tert-butyl) Benzoic Acid

Yield: 0.0556 g, (0.31 mmol quant.); white solid. m. p. = 145–147 °C. ^1^H NMR (500 MHz, CDCl_3_): δ = 8.05 (d, *J* = 8.4 Hz, 2 H), 7.50 (d, *J* = 8.4 Hz, 2 H), 1.35 (s, 9 H). ^13^C NMR (125 MHz, CDCl_3_): δ = 171.9, 157.7, 130.3, 126.6, 125.6, 35.3, 31.2. HRMS (DART): *m*/*z* [M + H]^+^ calcd for C_11_H_14_O_2_: 179.1067; found: 179.1060.

#### 3.2.12. [1,1′-Biphenyl]-4-carboxylic Acid

Yield: 0.0599 g, (0.30 mmol 99%); white solid. m. p. = 215–218 °C. ^1^H NMR (500 MHz, DMSO-d_6_): δ = 8.02 (d, *J* = 8.4 Hz, 2 H), 7.80 (d, *J* = 8.4 Hz, 2 H), 7.74 (d, *J* = 7.3 Hz, 2 H), 7.52–7.41 (m, 3 H). ^13^C NMR (125 MHz, DMSO-d6): δ = 167.1, 144.3, 139.0, 130.0, 129.6, 129.1, 128.3, 127.0, 126.8. HRMS (DART): *m*/*z* [M + H]^+^ calcd for C_13_H_10_O_2_: 199.0754; found: 199.0754.

## 4. Conclusions

In summary, we have studied the synthesis of ionic liquid-supported anthraquinone photocatalysts and demonstrated that they can be used in the oxidative cleavage of vicinal diols. This reaction system was effective in converting various diols to the corresponding benzoic acid derivatives. After completion of the reaction, purification steps, such as column chromatography, are not required because the product can be extracted in an organic solvent. Moreover, the reaction system that utilizes a (bmim)BF_4_ solution of the IL-AQ(BF_4_) ionic liquid-supported catalyst can be reused up to five times. This system is advantageous as it is environmentally friendly because it can be reused and utilizes UV light, and thus that does not create waste.

## Figures and Tables

**Table 1:** Optimization of the reaction conditions ^a^. ^a^ Condition:1-phenylethane-1,2-diol (0.3 mmol), photocatalyst (0.03 mmol), ionic liquid (3 mL), 80 °C, 15 h. ^b^ Isolated yield. ^c^ In the dark. ^d^ Amount of photocatalyst is 0.01 eq.

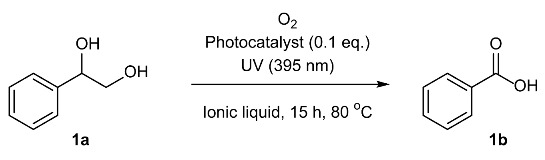			
Entry	Photocatalysts	Ionic Liquid	Yield ^b^ (%)
1	IL-AQ(PF_6_)	(bmim)PF_6_	96
2	IL-AQ(TFSI)	(bmim)TFSI	98
3	IL-AQ(BF_4_)	(bmim)BF_4_	quant.
4	IL-AQ(Br)	(bmim)Br	99
5	IL-AQ(PF_6_)	(bmim)BF_4_	88
6	IL-AQ(TFSI)	(bmim)BF_4_	91
7	IL-AQ(Br)	(bmim)BF_4_	60
8	anthraquinone	(bmim)BF_4_	98
9 ^c^	IL-AQ(BF_4_)	(bmim)BF_4_	n. r.
10	-	(bmim)BF_4_	40
11 ^d^	IL-AQ(BF_4_)	(bmim)BF_4_	65

**Table 2:** Scope of the vicinal diols ^a^. ^a^ Condition: substrate (0.3 mmol), IL-AQ(BF_4_) (0.03 mmol), (bmim)BF_4_ (3 mL), 80 °C, 15 h. Isolated yield.

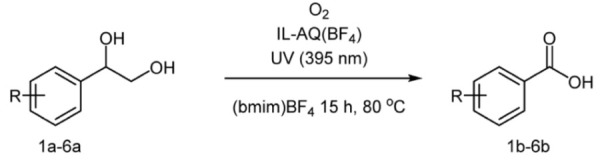		
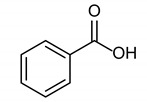	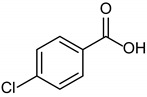	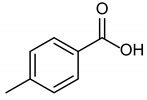
1b quant.	2b 98%	3b quant.
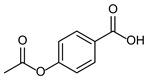	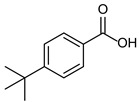	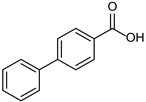
4b 95%	5b quant.	6b 99%

**Table 3:** Recycling of the ionic liquid-supported catalysts ^a^. ^a^ Conditions: 1-phenylethane-1,2-diol (0.3 mmol), photocatalyst (0.03 mmol), ionic liquid (3 mL), 80 °C, 15 h. Isolated yield.

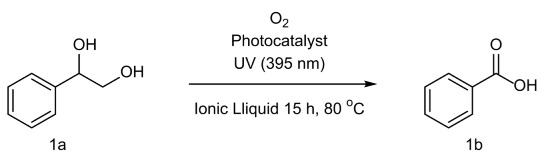						
Entry	Condition	1 Cycle	2 Cycles	3 Cycles	4 Cycles	5 Cycles
1	Photocatalyst; IL-AQ(BF_4_) Ionic Liquid; (bmim)BF_4_	quant.	quant.	quant.	99%	quant.
2	Photocatalyst; IL-AQ(PF_6_) Ionic Liquid; (bmim) PF_6_	96%	96%	90%	95%	97%
3	Photocatalyst; IL-AQ(TFSI) Ionic Liquid; (bmim) TFSI	98%	98%	72%	-	-

## Data Availability

The data presented in this study are available in Supplementary Material.

## Supplementary Materials

The following supporting information can be downloaded at: https://www.mdpi.com/article/10.3390/ijms24087141/s1.
