# Supplementary material for: Ionic Liquid-Supported Photocatalysts: A Reusable Environmentally Friendly Oxidation Reaction System That Uses Air and Light
Source: Int J Mol Sci. 2023 Apr 12;24(8):7141. doi: 10.3390/ijms24087141 (PMC10138590; doi:10.3390/ijms24087141)

Supporting Information

Ionic liquid-supported photocatalysts.
A reusable environ-mentally friendly oxidation reaction system that uses air and light.

Shinichi Koguchi*, Haruto Fujita and Yuga Shibuya

Department of Chemistry, Tokai University,
4-1-1 Kitakaname, Hiratsuka-shi, Kanagawa,259-1292 Japan.
E-mail: koguchi@tokai-u.jp

1H, and 13C, NMR spectra of Ionic liquid-supported catalysts.

^{1}H NMR of 2-bromomethyl-9,10-anthraquinone

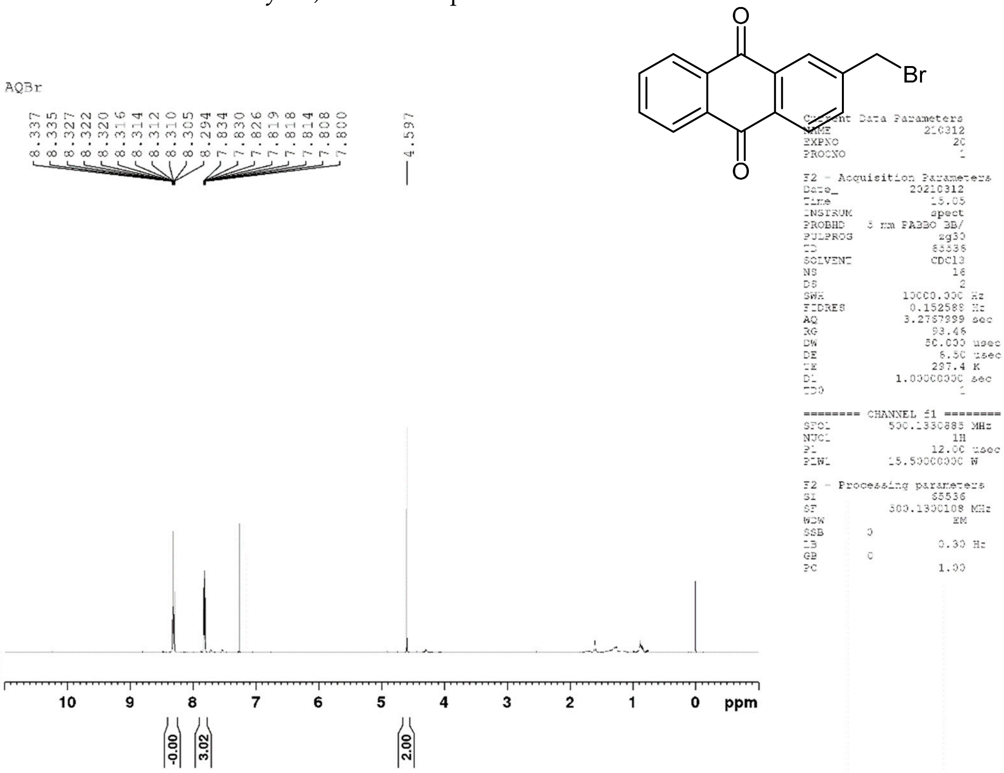

^{13}C NMR of 2-bromomethyl-9,10-anthraquinone

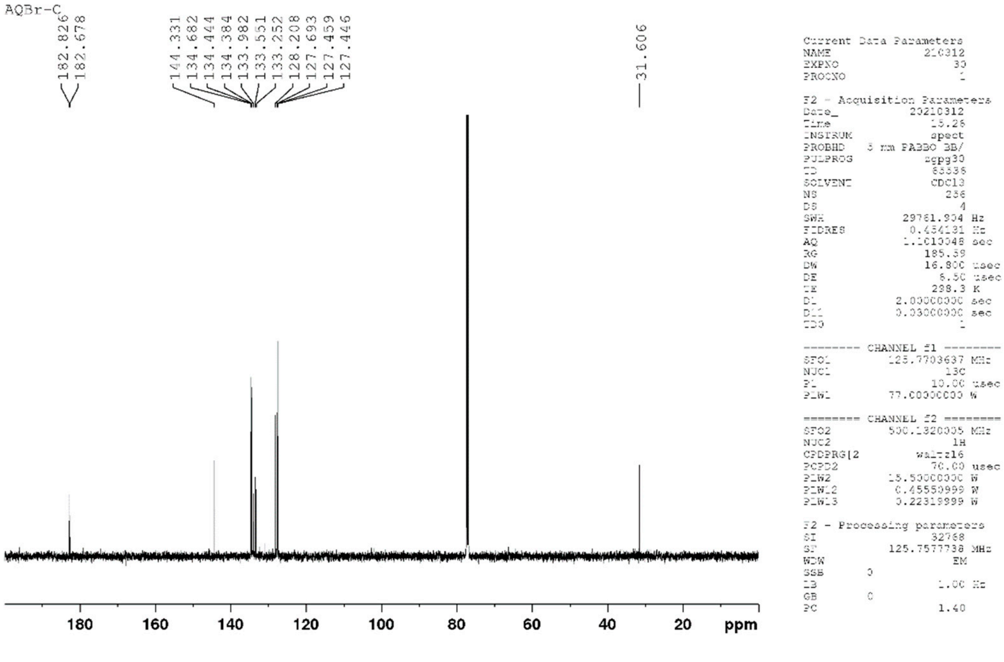

^{1}H NMR of 1-((9,10-Anthraquinon-2-yl) methyl)-3-methyl-1H-imidazol-3-ium Bromide IL-AQ(Br)

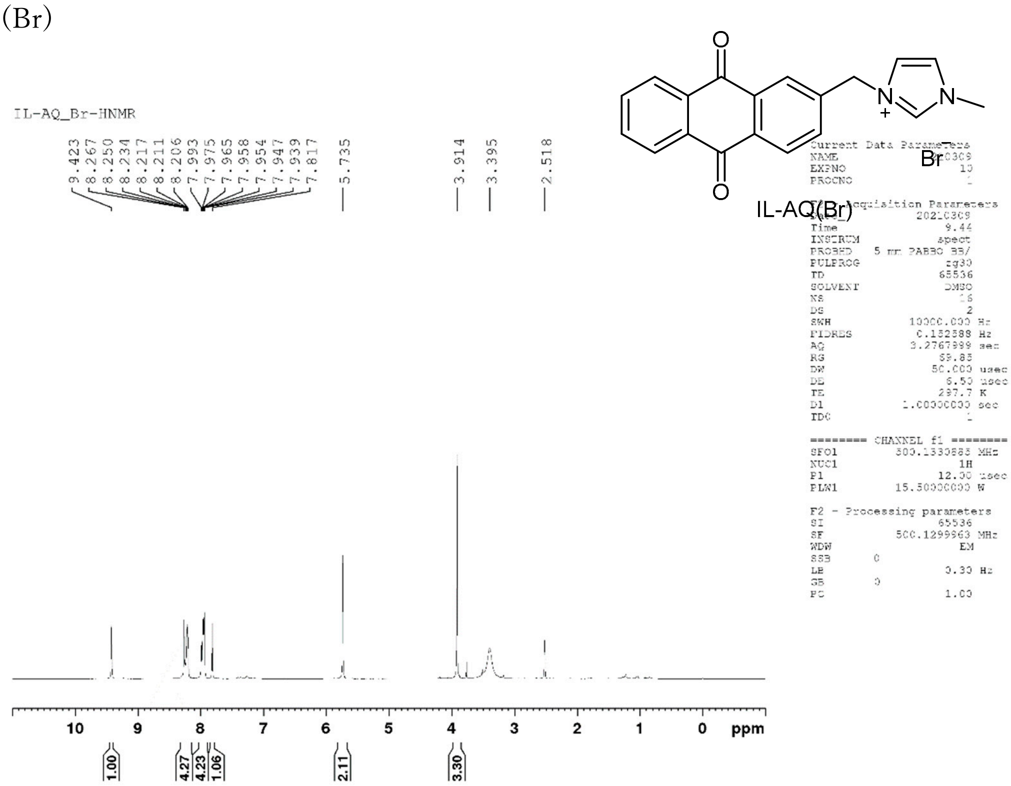

^{13}C NMR of 1-((9,10-Anthraquinon-2-yl) methyl)-3-methyl-1H-imidazol-3-ium Bromide IL-AQ(Br)

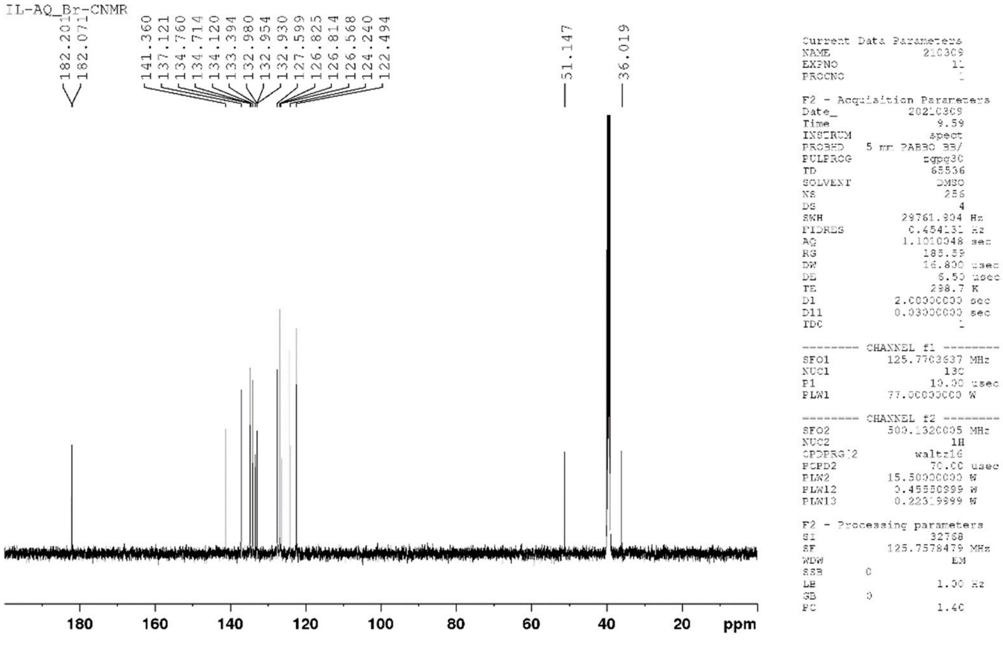

^{1}H NMR of 1-((9,10-Anthraquinon-2-yl) methyl)-3-methyl-1H-imidazol-3-ium Bis(trifluoromethanesulfonyl) imide IL-AQ (TFSI)

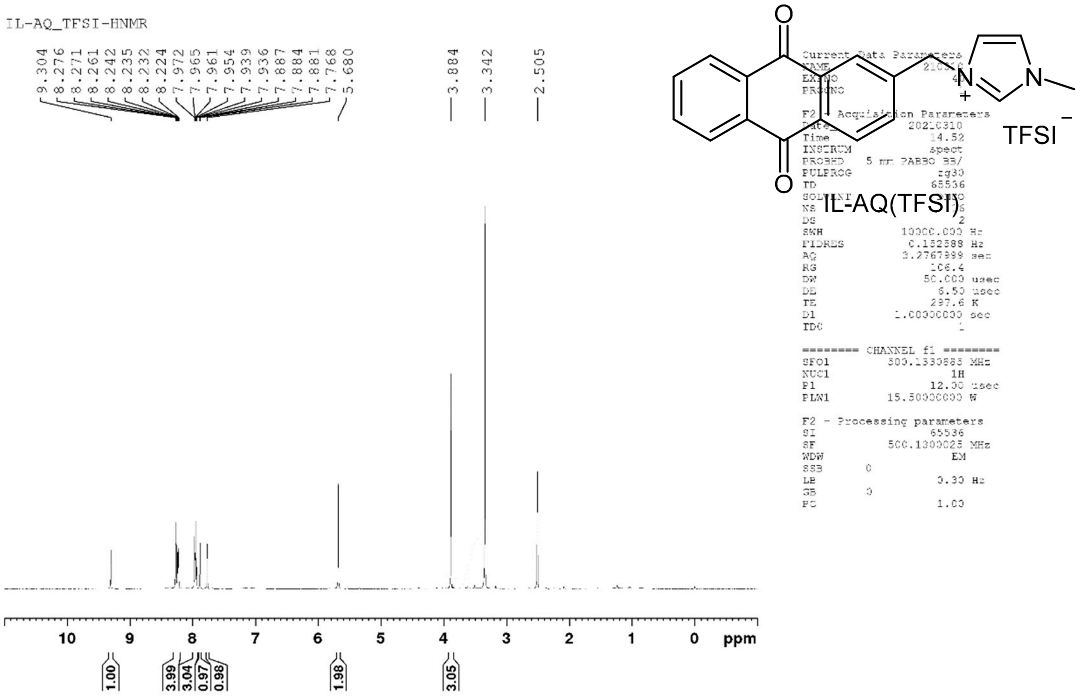

^{13}C NMR of 1-((9,10-Anthraquinon-2-yl) methyl)-3-methyl-1H-imidazol-3-ium Bis(trifluoromethanesulfonyl) imide IL-AQ (TFSI)

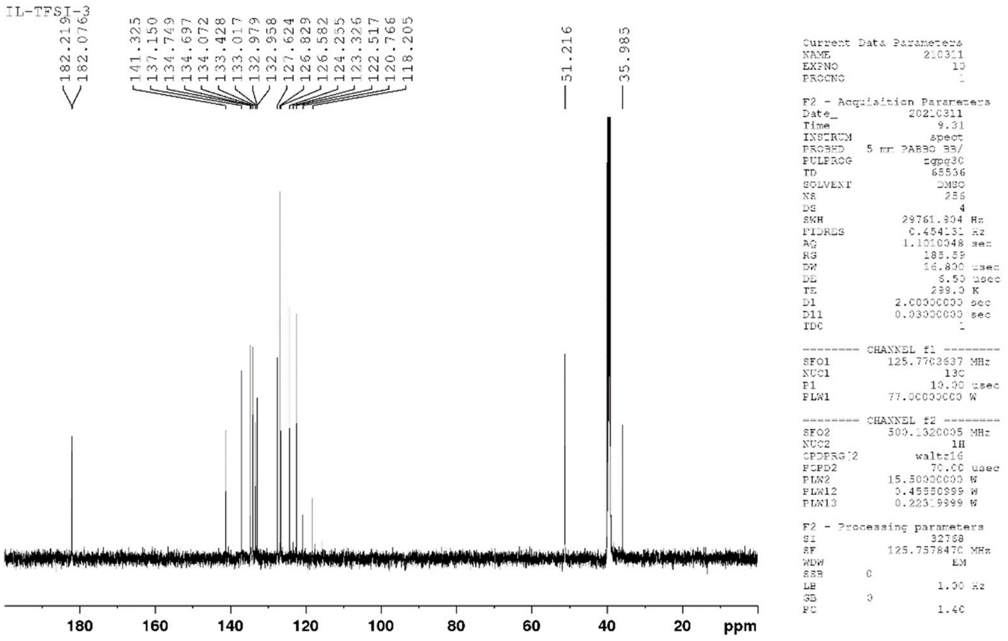

^{1}H NMR of 1-((9,10-anthraquinon-2-yl)methyl)-3-methyl-1H-imidazol-3-ium Tetrafluoroborate IL-AQ (BF_4)

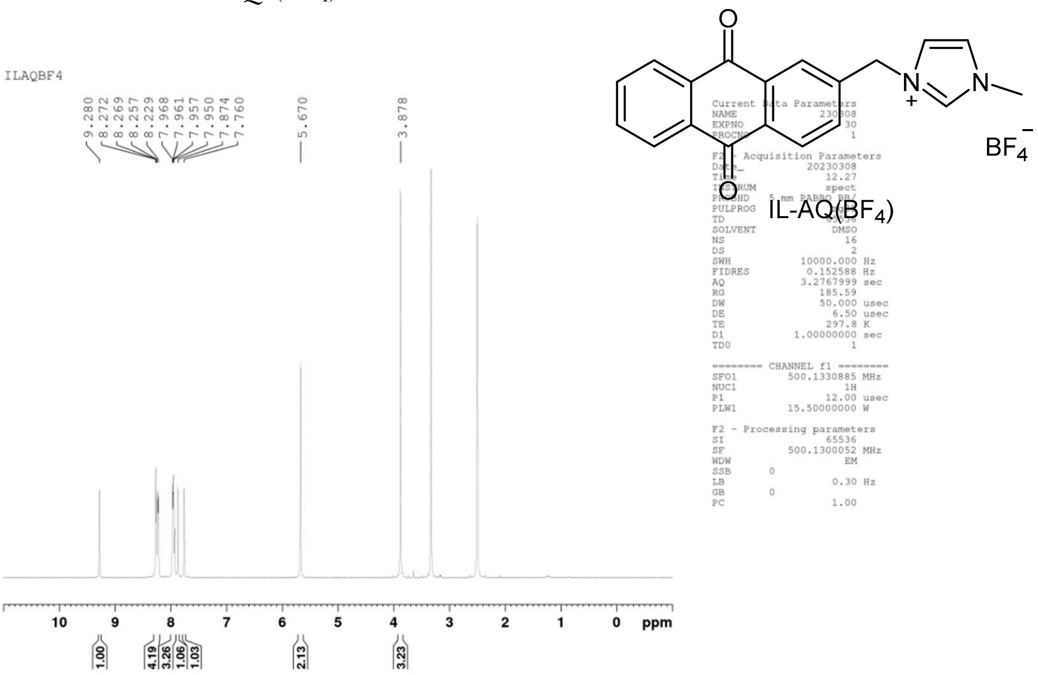

^{13}C NMR of 1-((9,10-anthraquinon-2-yl)methyl)-3-methyl-1H-imidazol-3-ium Tetrafluoroborate IL-AQ (BF_4)

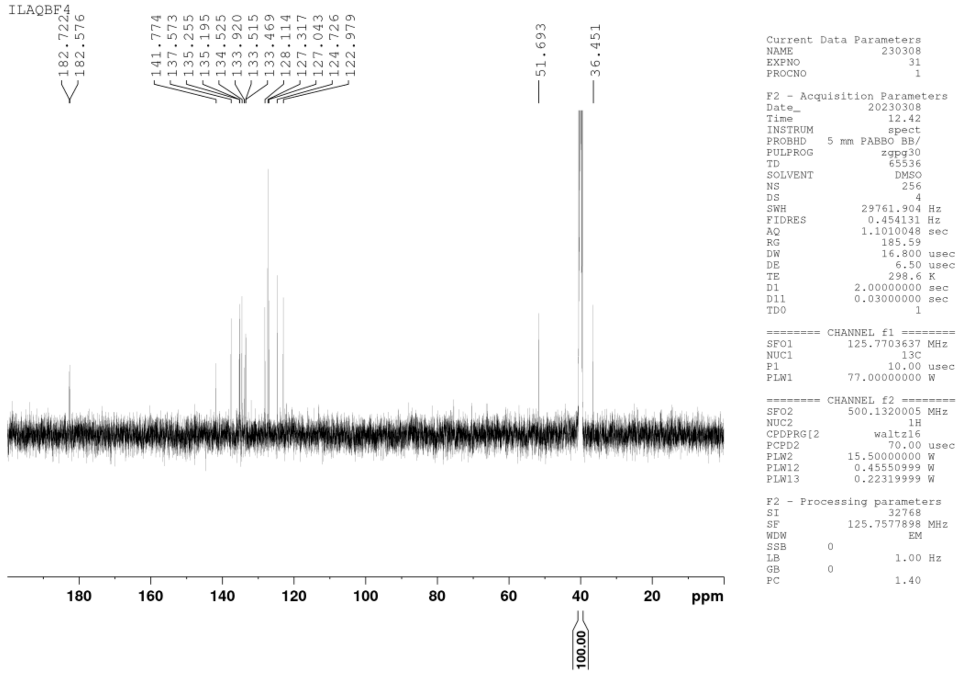

^{1}H NMR of 1-((9,10-anthraquinon-2-yl) methyl)-3-methyl-1H-imidazol-3-ium Hexafluorophosphate IL-AQ (PF_6)

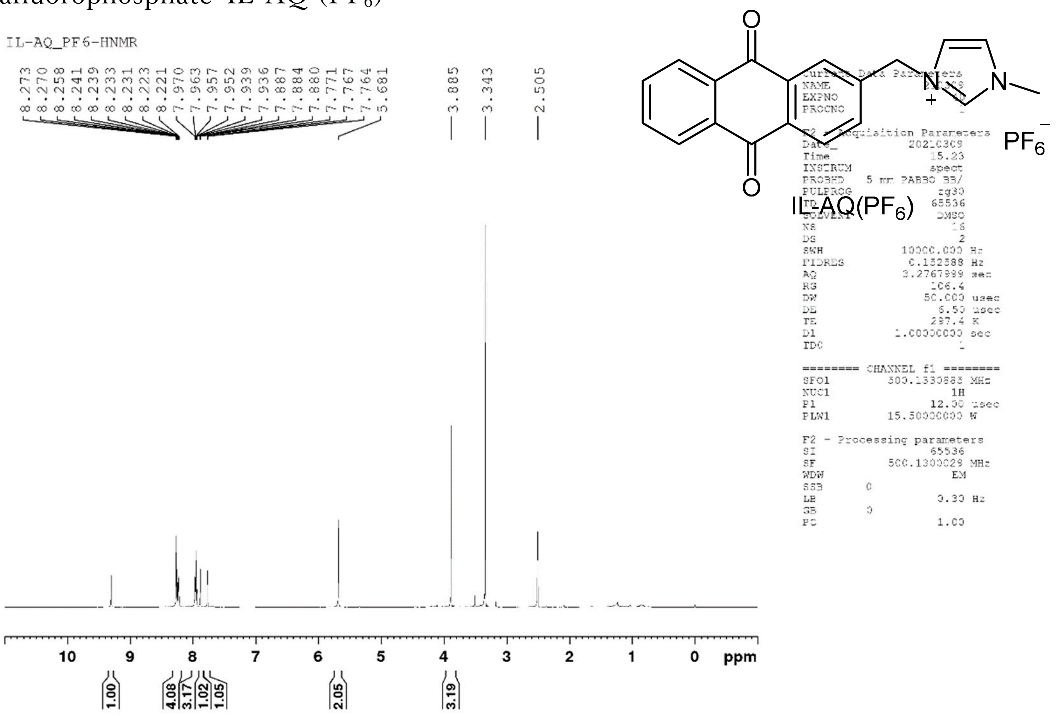

^{13}C NMR of 1-((9,10-anthraquinon-2-yl) methyl)-3-methyl-1H-imidazol-3-ium Hexafluorophosphate IL-AQ (PF_6

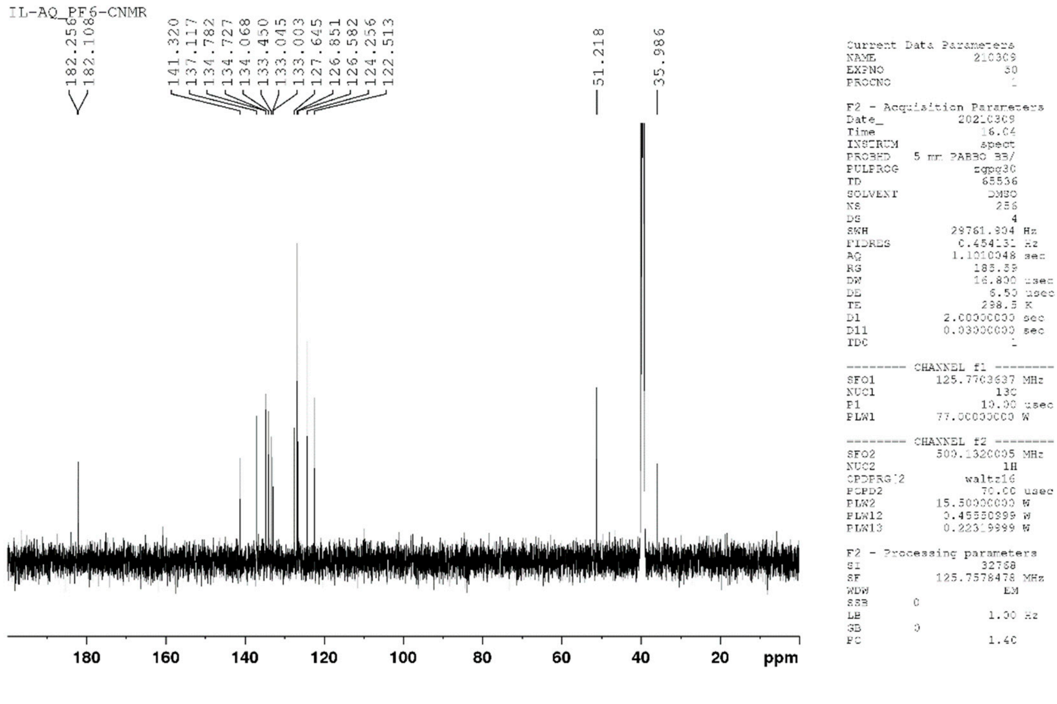

^{1}H NMR of Benzoic Acid

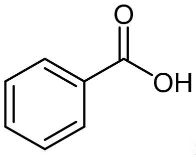

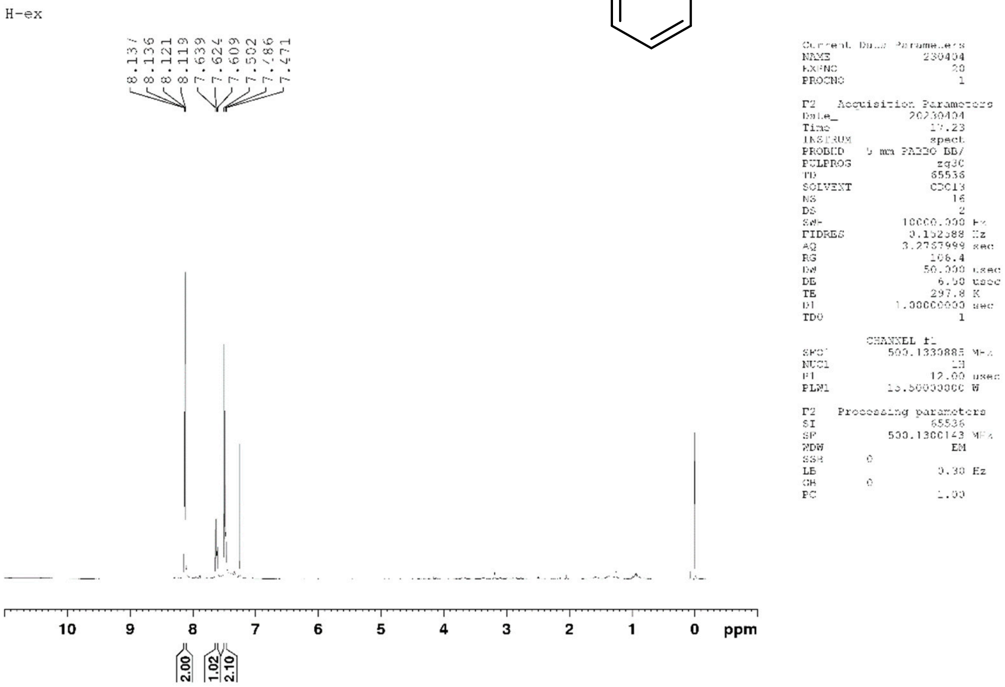

^{13}C NMR of Benzoic Acid

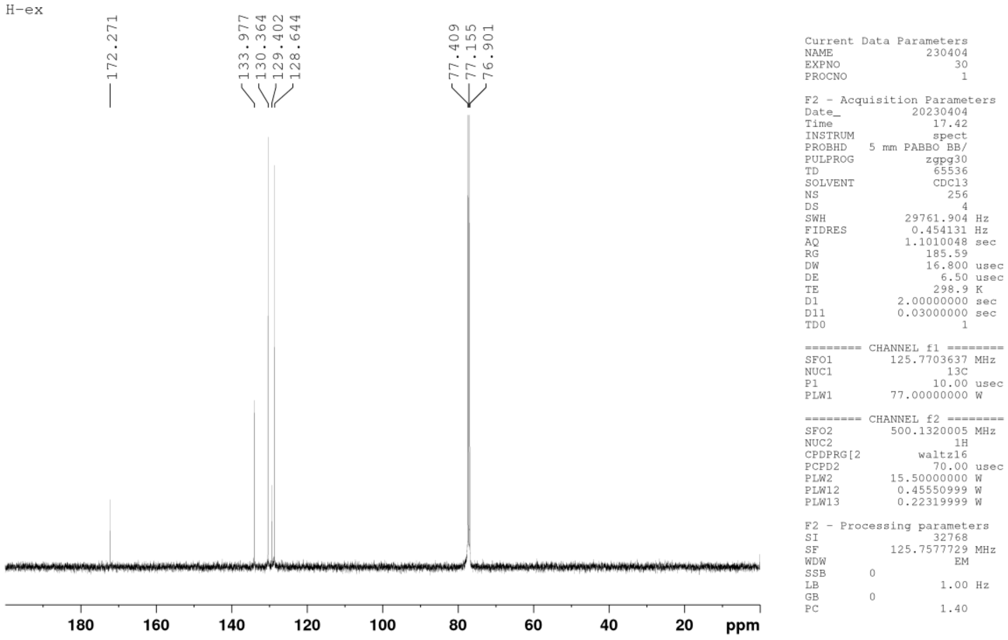

^{1}H NMR of 4-chlorobenzoic acid

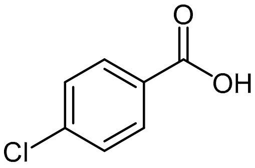

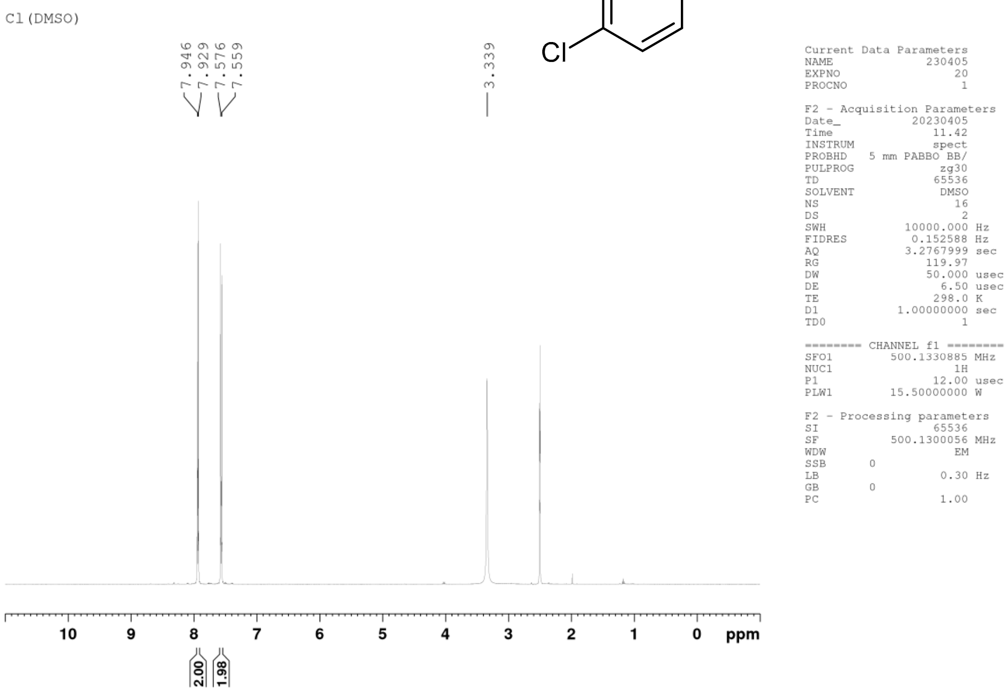

^{13}C NMR of 4-chlorobenzoic acid

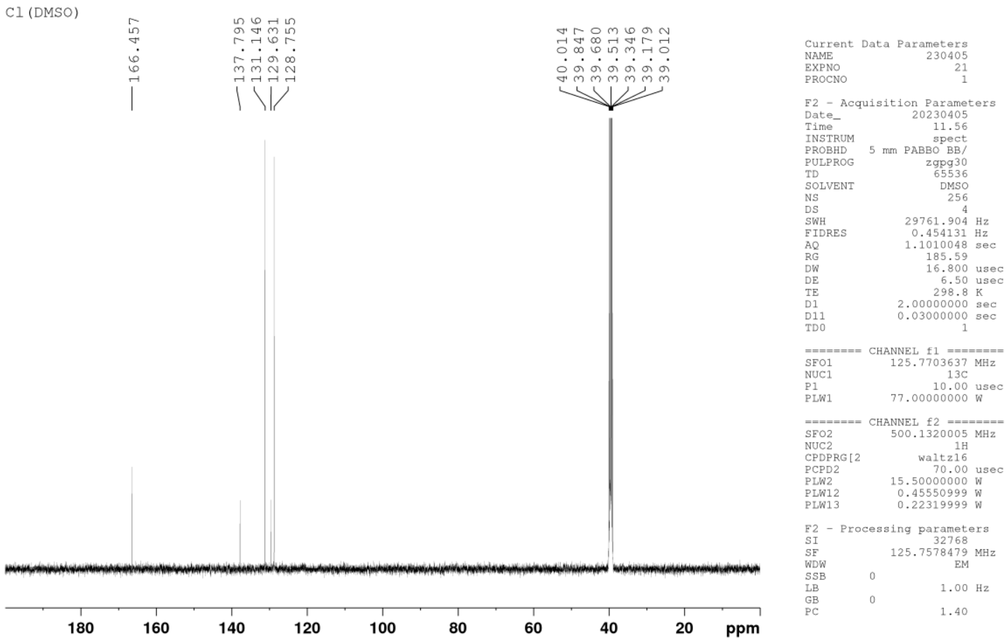

^{1}H NMR of 1H NMR of 4-methylbenzoic acid

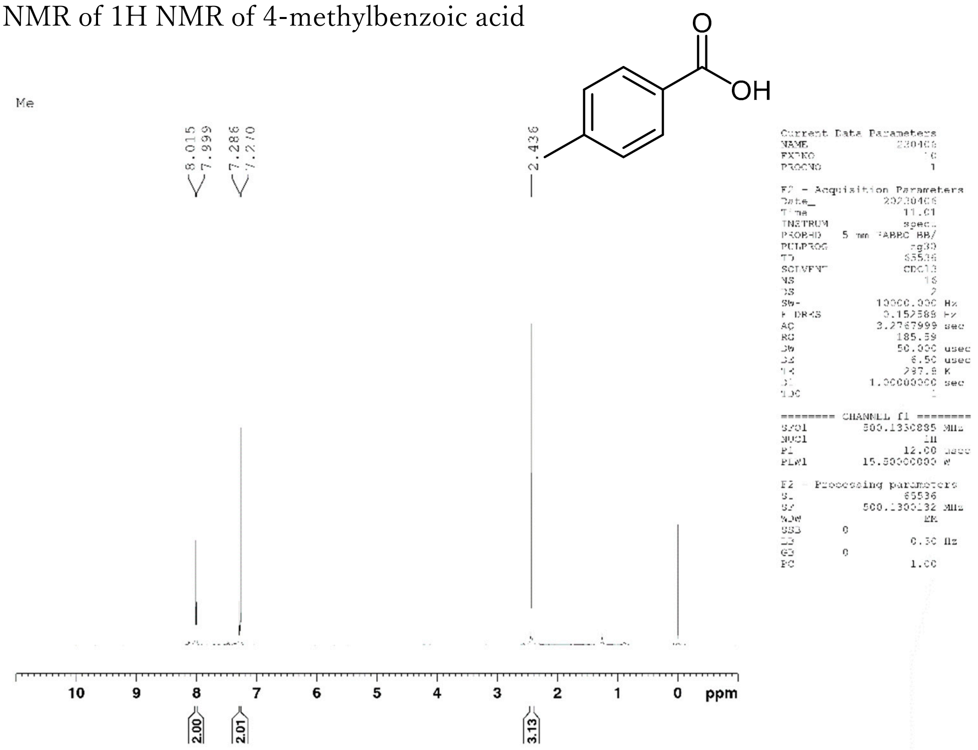

^{13}C NMR of 4-methylbenzoic acid

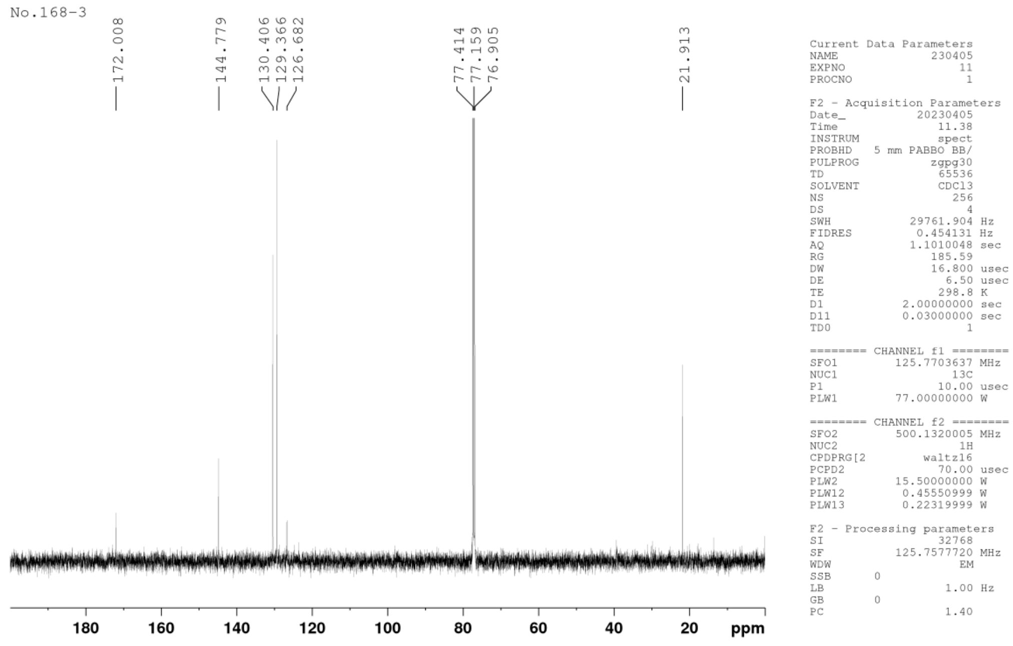

^{1}H NMR of 1H NMR of 4-acetoxybenzoic acid

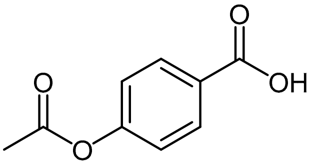

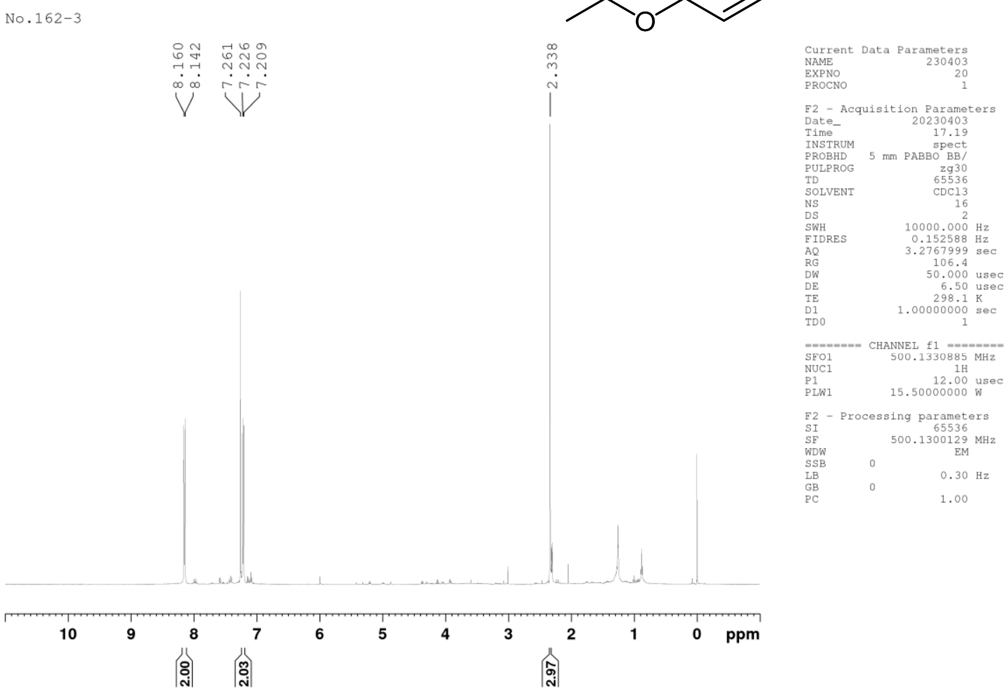

^{13}C NMR of 4-acetoxybenzoic acid

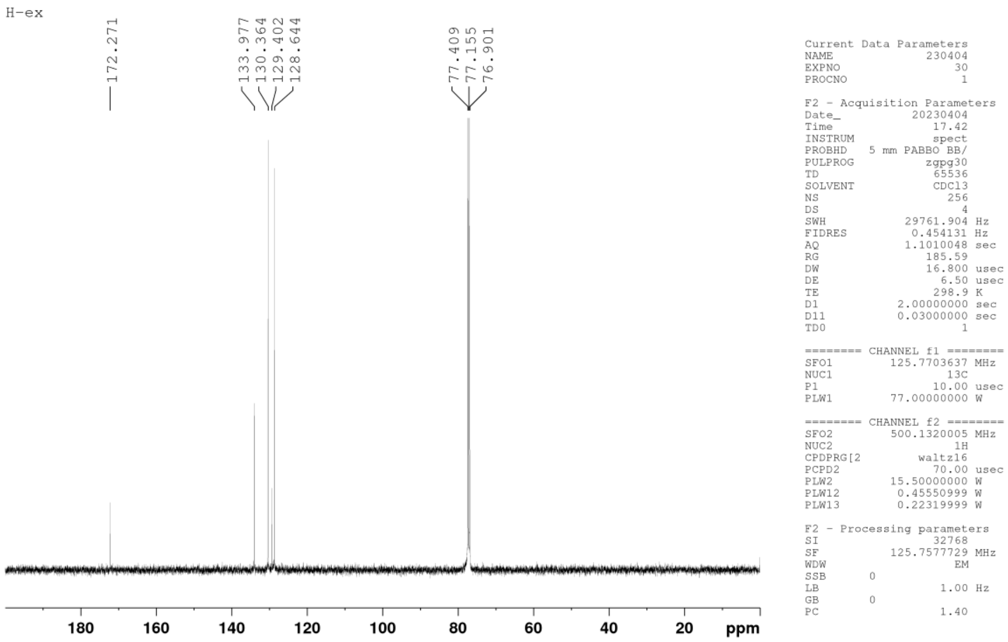

^{1}H NMR of 1H NMR of 4-(*tert*-butyl) benzoic acid

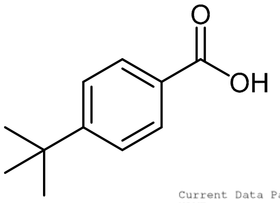

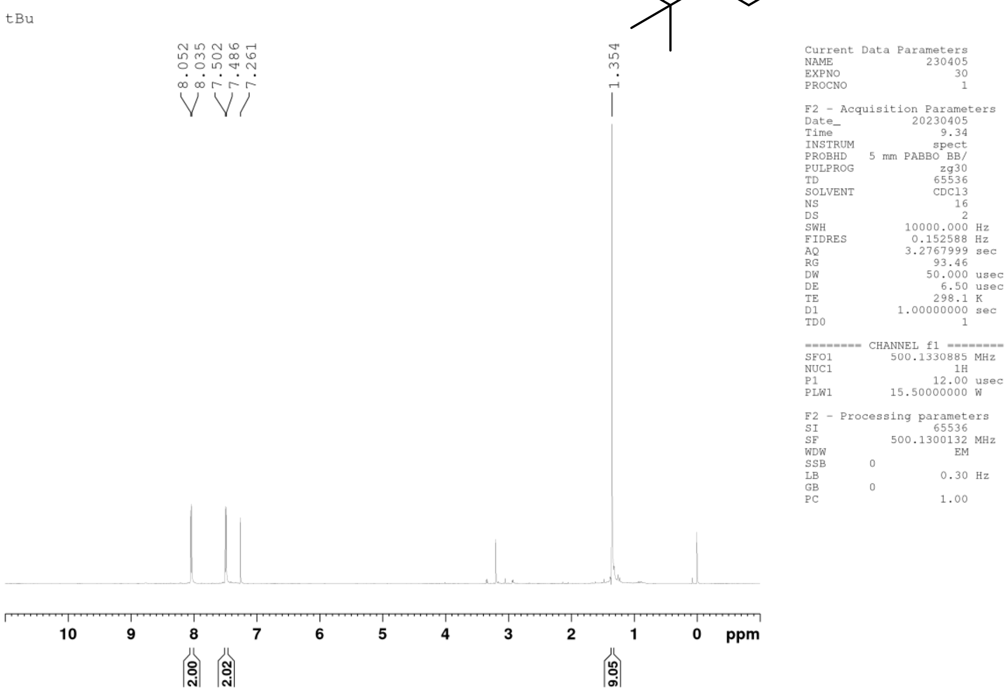

^{13}C NMR of 4-(*tert*-butyl) benzoic acid

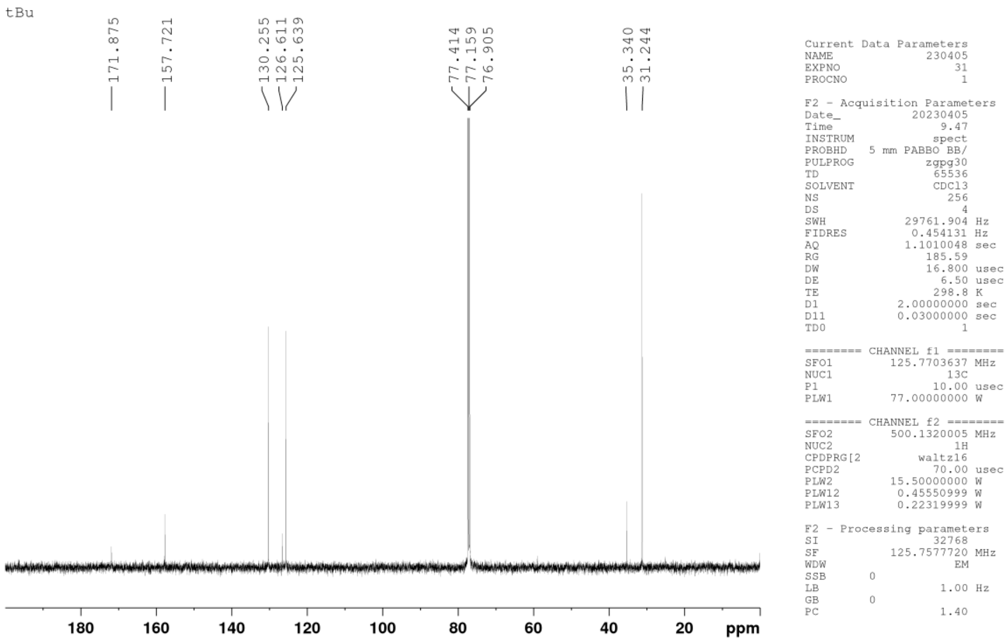

^{1}H NMR of 1H NMR of [1,1'-biphenyl]-4-carboxylic acid

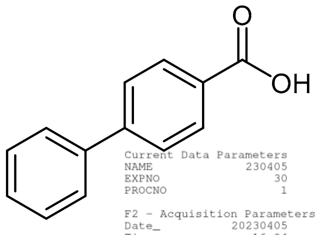

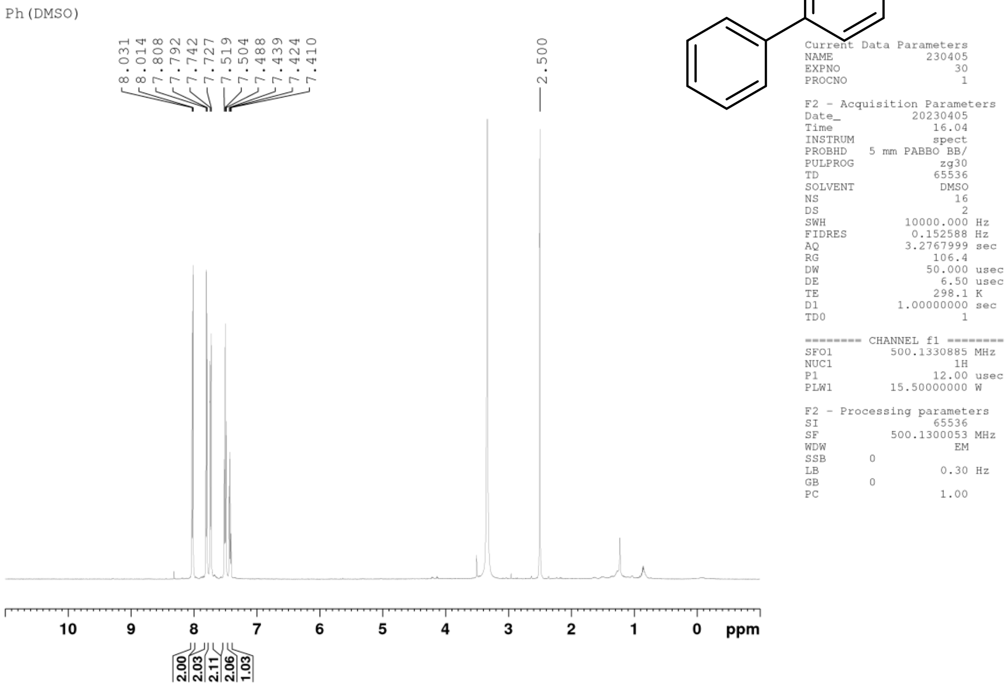

^{13}C NMR of [1,1'-biphenyl]-4-carboxylic acid

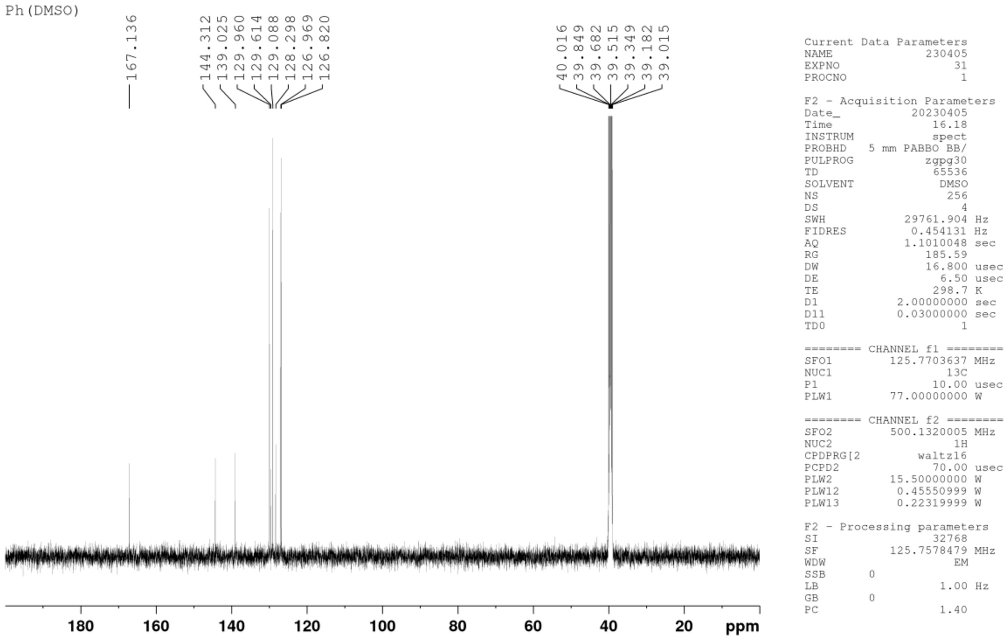

Supplement: Supplementary file 1 [file ijms-24-07141-s001.zip › ijms-2305428-supplementary.pdf]
